# Human Gyrovirus DNA in Human Blood, Italy

**DOI:** 10.3201/eid1806.120179

**Published:** 2012-06

**Authors:** Fabrizio Maggi, Lisa Macera, Daniele Focosi, Maria Linda Vatteroni, Ugo Boggi, Guido Antonelli, Marc Eloit, Mauro Pistello

**Affiliations:** Pisa University Hospital, Pisa, Italy (F. Maggi, L. Macera, D. Focosi, M.L. Vatteroni, U. Boggi, M. Pistello);; “Sapienza” University of Rome, Rome, Italy (G. Antonelli); Institut Pasteur, Paris, France (M. Eloit);; Pathoquest, Paris (M. Eloit)

**Keywords:** human gyrovirus, HGyV DNA, transplantation, HIV, real-time PCR, sequencing, viruses, Italy, blood

## Abstract

Human gyrovirus (HGyV) is a recent addition to the list of agents found in humans. Prevalence, biologic properties, and clinical associations of this novel virus are still incompletely understood. We used qualitative PCRs to detect HGyV in blood samples of 301 persons from Italy. HGyV genome was detected in 3 of 100 solid organ transplant recipients and in 1 HIV-infected person. The virus was not detected in plasma samples from healthy persons. Furthermore, during observation, persons for whom longitudinal plasma samples were obtained had transient and scattered presence of circulating HGyV. Sequencing of a 138-bp fragment showed nucleotide identity among all the HGyV isolates. These results show that HGyV can be present in the blood of infected persons. Additional studies are needed to investigate possible clinical implications.

## Abstract 2

HGyV in blood suggests the infection might be systemic.

In 2011, Sauvage et al. reported the discovery of a novel virus in human skin specimens and named it human gyrovirus (HGyV) (*1*). The characteristics of its genome—a single, closed molecule of circular, negative-sense DNA ≈2,300 nt long—and sequence homology with the chicken anemia virus (CAV) have suggested that HGyV might be the first human-infecting member of the genus *Gyrovirus*, which is part of the family *Circoviridae* and encompasses only 1 previously known species, CAV (*2*).

The genome of HGyV, which resembles CAV more closely than other members of the family (*1**,**3*), contains an untranslated region of ≈380 nt and 3 major partially overlapping open reading frames (named viral protein [VP] 1, VP2, and VP3) that encode proteins of 465, 231, and 31 aa, respectively. Whether HGyV and equivalent CAV proteins have similar functions is unknown. VP3 products of HGyV and CAV (for which the coded protein has been named apoptin) share short, functionally pivotal amino acid motifs, suggesting that HGyV also encodes an apoptin-like protein. The CAV apoptin induces tumor-specific apoptosis in a p53-independent fashion and has been shown to be a potential anticancer therapeutic agent in various animal models (*4**–**8*).

The epidemiology, biologic properties, and pathogenic potential of HGyV remain poorly understood. Sauvage et al. (*1*) detected the HGyV genome in nonlesional skin specimens of healthy persons and 1 HIV-positive patient but not in respiratory and fecal samples. This observation suggests that HGyV is most likely part of the normal skin microflora of humans, similarly to other recently discovered viruses (*9**,**10*). However, like related animal viruses, CAV infects a large range of cell types and causes a variety of pathologies (including bone marrow aplasia leading to aplastic anemia, hemorrhage, and lymphoid depletion) and increased death in young chicken (*11*). Also, CAV infection has been associated with the worsening of pathologies caused by other viral and bacterial agents (*11**–**13*).

Thus, because HGyV might cause clinically relevant disorders, guidance in choosing the directions for clinical investigation is crucial and needs to come from studies aimed at defining the prevalence of HGyV infection in different human populations, portal of entry, type of cells targeted during primary amplification, and site of latency/persistence. We investigated the presence of HGyV DNA in blood samples of 301 persons in Italy using specific PCRs. The results indicated overall HGyV positivity of 1.3%.

## Materials and Methods

### Patients and Samples

During December 2011, we studied 301 randomly selected persons living in central Italy. Most (251) were diseased patients whose blood samples had been submitted to the Virology Unit, Pisa University Hospital (Pisa, Italy), by local hospitals for routine virologic analysis; the remaining 50 were healthy blood donors. The patients comprised 151 HIV-infected persons (mean ± SD age 47 ± 14 years [range 18–80 years]; 115 men) who, before initiation of the study, had received no antiretroviral treatment. The patients also were examined for xenotropic murine-leukemia virus-related virus in a previous study (*14*). The other 100 patients (mean ± SD age 56 ± 8 years [range 36–69 years]; 71 men) were solid organ transplant recipients: 50 had received a liver transplant, and 50 had received a kidney transplant. Plasma samples were collected from patients on the day of transplant and then at selected times after transplant. Aliquots were prepared immediately, stored, and kept under sterile conditions at –80°C until use. Written informed consent was collected from each patient.

### HGyV DNA Detection

Viral DNA was extracted from 400 µL of peripheral whole blood or 200 µL of plasma by using the Maxwell 16 System (Promega, Madison, WI, USA) or QIAamp DNA blood kit (QIAGEN, Hilden, Germany), respectively, according to the manufacturers’ instructions. Extracted DNA was amplified with 2 PCR protocols (developed and provided by V. Sauvage et al.), which target the VP1 gene of the viral genome.

The first amplification was a single-step TaqMan real-time PCR (rtPCR) designed on a 72-nt fragment. The assay was performed in a 25-µL volume containing 400 nmol/L of each primer (HGyV-rtFP: 5′-TGCTTGCAACAATGCCTTTAGA-3′; HGyV-rtRP: 5′-CCCTGCAAGTGCTGAGGATAA-3′), 200 nmol/L double-labeled probe (HGyV-rtP: 5′-FAM-CAAAGAGCAAAATCGGAGGCCCTAACC-TAMRA-3′) 5 µL extracted viral DNA, and the Universal PCR Master Mix (Applied Biosystems, Foster City, CA, USA) containing deoxynucleoside triphosphate, and Taq DNA polymerase. Reaction was run in triplicate for each sample in an iQCYCLER rtPCR detection instrument (Bio-Rad Laboratories, Hercules, CA, USA) by using a standardized program (95°C 10 min; 45 cycles of 15 s at 95°C, and 60°C 60 s; and 40°C 30 s). A sample was considered rtPCR positive when HGyV DNA was detected in 2 of 3 replicas and when amplifications were specific as determined by 2% agarose gel electrophoresis.

A 178-bp fragment was amplified by a nested PCR format, described by Sauvage et al. (*1*) with modifications. This PCR was performed for 25 cycles with sense primer HGyV-OF (5′-CAAAATCGGAGGCCCTAACCC-3′) and antisense primer HGyV-OR (5′-ATGCCTGAATAGCTGCCAGCC-3′) under the following conditions: denaturation at 94°C for 60 s, annealing at 55°C for 60 s, and extension at 72°C for 45 s. The product of this reaction (10 µL) was then re-amplified for 35 cycles with internal primers HGyV-IF (5′-GGTCAGCACAAACGACGCAG-3′) and HGyV-IR (5′- AGGTCTCCCATAGCGTCCAG-3′) at the same PCR conditions. The reactions were conducted in a 50-µL PCR mixture containing Taq DNA polymerase, each deoxynucleoside triphosphate at a concentration of 10 mmol/L, primers (20 µmol/L each), and optimized buffer components.

All samples were tested at least in duplicate and on different occasions. The amplified product was analyzed by electrophoresis on a 2% agarose gel after ethidium bromide staining. Amplicon size was compared with standard molecular mass markers. To minimize contamination risk, serum handling, DNA extraction, PCR amplification, and electrophoresis analysis were conducted in separated rooms. Negative controls were added during DNA extraction and PCR amplification. To validate the amplification process, positive controls (obtained from M.E.) were run in each PCR.

### Sequence Analysis

All HGyV PCR–positive isolates were characterized by sequencing a 138-bp fragment (from nt 1328 to 1465 of VP1 gene of the representative isolate FR823283) encompassing the target region of nested PCR. PCR amplicons, purified from the gel by using a QIAquick Gel Extraction Kit (QIAGEN, Chatsworth, CA, USA), were sequenced by using the Big Dye Terminator Cycle Sequencing Kit (Applied Biosystems) and an automatic DNA sequencer (ABI model 3130; Applied Biosystems). Nucleotide sequences were aligned with the only sequence available at GenBank at the time of writing and by using the ClustalW algorithm included in BioEdit version 7.0.9.0 (www.clustal.org).

## Results

### Evidence of HGyV Infections

No samples from 50 healthy blood donors studied yielded positive results for HGyV (Table 1). Of 4 samples in which HGyV DNA was detected, 3 (6%) were from kidney transplant recipients and 1 (0.7%) was from an HIV-infected patient.

**Table 1:** Prevalence of human gyrovirus DNA in 251 HIV-positive or transplant recipient patients and 50 blood donors, Italy

Group	Material tested	No. persons	No. (%) HGyV positive
Healthy persons	Plasma	50	0
HIV-positive patients	Whole blood	151	1 (0.7)
Organ transplant recipients			
Liver	Plasma	50	0
Kidney	Plasma	50	3 (6.0)
Total		301	4 (1.3)

### Longitudinal Study of HGyV Viremia in Transplant Recipients

We examined plasma samples from 100 transplant patients for whom we had sequential samples obtained at selected times after transplant. Three of these patients who had received a kidney transplant tested positive for HGyV DNA, indicating that they had systemic HGyV infection. When additional samples of these patients were examined, a similar pattern emerged (Table 2). Plasma samples from 2 transplant recipients were already HGyV positive when they were first examined before transplantation. Subsequently, HGyV DNA detection was intermittent in the posttransplant samples: it was positive at month 12 (patient AL) and 6 (patient CV) but negative in the other samples tested. For patient MG, we examined 4 blood samples obtained before and after HGyV detection in plasma. At all these times, the plasma tested repetitively negative for HGyV DNA.

**Table 2:** Time course of human gyrovirus DNA detection in 3 kidney transplant patients, Italy*

Months after transplant	Plasma HGyV DNA		
	Patient AL	Patient CV	Patient MG
0	Positive	Positive	Negative
1	ND	Negative	Negative
3	ND	ND	Negative
6	Negative	Positive	Positive
9	ND	Negative	Negative
12	Positive†	Negative	ND
15	Negative	ND	ND
*HGyV, human gyrovirus; ND, not determined. †The urine sample tested at month 12 was HGyV negative.			

### Genetic Analysis of HGyV Isolates

**Figure A1:**
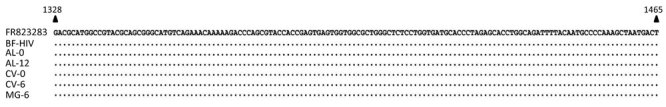
Alignment of the nucleotide sequences of the human gyrovirus genome on the basis of a 138-bp segment (nt 1328–1465) of the viral protein 1 gene. The sequences of the isolate from France (FR823283) and of the 6 isolates found in this study are shown. Nucleotide positions are according to isolate FR823283 (*1*). Hyphens indicate identity with the prototype sequence of isolate FR823283 (*1*).

Sequencing was conducted on all 6 PCR fragments obtained. All the isolates were related to the previously published strain, and the sequences obtained were virtually identical in the nucleotide fragment examined (Figure A1). When blood specimens from the same patient were sequenced, no nucleotide change was noted among the viral sequence fragments obtained at any time.

## Discussion

The recent discovery of a human virus similar to CAV prompted an investigation of samples collected from patients and from healthy blood donors in central Italy. This investigation confirms that HGyV is present in humans, extends the previous findings, and raises several points. In the only study published, the HGyV genome had been investigated in 115 nonlesional skin specimens from adults and 138 specimens (46 nasopharyngeal aspirates and 92 fecal samples) from children. HGyV DNA was found in only 5 nonlesional skin specimens (*1*), suggesting that the virus could be a member of the human skin virome. In our study, plasma samples were taken from 251 immunocompromised patients (151 patients with HIV infection and 100 transplant recipients) and 50 healthy donors. HGyV DNA was demonstrated in the plasma of 4 persons, all with dysfunctional immune systems.

The presence of HGyV in blood of infected humans suggests that the infection might also be systemic. The finding is not totally unexpected because CAV and the recently discovered avian gyrovirus 2, a virus genetically similar to HGyV, can circulate in the blood of infected animals (*15**–**18*). The clinical significance of HGyV viremia and relationship to induction of pathogenic processes is unclear. However, among the patients in whom virus was demonstrated, most had received a kidney transplant and thus had severe underlying nephropathy. The 3 kidney transplant recipients were a 52-year-old man with focal segmental glomerulosclerosis, a 21-year-old woman with lacrimoauriculodentodigital syndrome (i.e., Levy-Hollister syndrome), and a 57-year-old man with end-stage renal failure of unknown cause. All 3 recipients received basiliximab induction and triple maintenance immunosuppressive therapy with prednisone, mycophenolate mofetil, and cyclosporine A. The remaining HGyV-positive patient was a 32-year-old HIV-positive man who, when tested for HGyV, had an HIV load of 156,000 copies/mL and a CD4+ count of 465 cells/µL.

Data collected over time showed the occasional detection of viral DNA in blood. In fact, a similar pattern was observed in all the positive patients studied: plasma HGyV-positive samples alternated with virus-negative samples, indicating that circulating virus was intermittent. This finding also was confirmed in the HIV-positive patient, for whom the only additional plasma sample obtained 18 months after HGyV detection was HGyV negative (data not shown). Analysis of more data from additional studies is needed to understand the role of this transient detection of the virus, which might represent a putative short-lived acute infection with possible subsequent re-infection or just declines of the HGyV load under the lower limit of sensitivity of the detection methods used.

The limited size of the PCR fragment sequenced does not enable us to determine with certainty whether the HGyV DNA detected before transplantation and at later times were the same. This information could explain possible reinfections and/or persistence of the virus. Further molecular studies with larger fragments from variable regions of HGyV genome will be necessary to evaluate whether the virus persists in the infected host.

## References

[1] Sauvage V, Chevalb J, Foulongnec V, Gouilha MA, Parientea K, Manuguerra JC, Identification of the first human gyrovirus, a virus related to chicken anemia virus. J Virol. 2011;85:7948–50. 10.1128/JVI.00639-11 21632766 PMC3147908

[2] Biagini P, Bendinelli M, Hino S, Kakkola L, Mankertz A, Niel C, Circoviridae. In: King AMQ, Adams MJ, Carstens EB, Lefkowitz EJ, editors. Virus taxonomy. Ninth report of the International Committee for the Taxonomy of Viruses. New York: Academic Press; 2012. p. 343–9.

[3] Schat KA. Chicken anemia virus. Curr Top Microbiol Immunol. 2009;331:151–83. 10.1007/978-3-540-70972-5_10 19230563

[4] Los M, Panigrahi S, Rashedi I, Mandal S, Stetefeld J, Essmann F, Apoptin, a tumor-selective killer. Biochim Biophys Acta. 2009;1793:1335–42. 10.1016/j.bbamcr.2009.04.002 19374922

[5] Noteborn MHM. Chicken anemia virus induced apoptosis: underlying molecular mechanisms. Vet Microbiol. 2004;98:89–94. 10.1016/j.vetmic.2003.10.003 14741120

[6] Zhang YH, Leliveld SR, Kooistra K, Molenaar C, Rohn JL, Tanke HJ, Recombinant apoptin multimers kill tumor cells but are nontoxic and epitope-shielded in a normal-cell–specific fashion. Exp Cell Res. 2003;289:36–46. 10.1016/S0014-4827(03)00188-5 12941602

[7] Danen-van Oorschot AAAM, van der Eb AJ, Noteborn MHM. The chicken anemia virus–derived protein apoptin requires activation of caspases for induction of apoptosis in human tumor cells. J Virol. 2000;74:7072–8. 10.1128/JVI.74.15.7072-7078.2000 10888647 PMC112225

[8] Kucharski TJ, Gamache I, Gjoerup O, Teodoro JG. DNA damage response signaling triggers nuclear localization of the chicken anemia virus protein apoptin. J Virol. 2011;85:12638–49. 10.1128/JVI.05009-11 21937663 PMC3209404

[9] Moens U, Ludvigsen M, Van Ghelue M. Human polyomaviruses in skin diseases. Patholog Res Int. 2011;2011:123491. 21941687 10.4061/2011/123491 PMC3173887

[10] Osiowy C, Sauder C. Detection of TT virus in human hair and skin. Hepatol Res. 2000;16:155–62. 10.1016/S1386-6346(99)00046-7

[11] Todd D. Circoviruses: immunosuppressive threats to avian species: a review. Avian Pathol. 2000;29:373–94. 10.1080/030794500750047126 19184829

[12] Haridy M, Goryo M, Sasaki J, Okada K. Pathological and immunohistochemical study of chickens with co-infection of Marek‘s disease virus and chicken anaemia virus. Avian Pathol. 2009;38:469–83. 10.1080/03079450903349162 19937536

[13] Toro H, van Santen VL, Hoerr FJ, Breedlove C. Effects of chicken anemia virus and infectious bursal disease virus in commercial chickens. Avian Dis. 2009;53:94–102. 10.1637/8408-071408-Reg.1 19432010

[14] Maggi F, Focosi D, Lanini L, Sbranti S, Mazzetti P, Macera L, Xenotropic murine leukaemia virus–related virus is not found in peripheral blood cells from treatment-naive human immunodeficiency virus–positive patients. Clin Microbiol Infect. 2012;18:184–8. 10.1111/j.1469-0691.2011.03580.x 21672082

[15] Tan J, Tannock GA. Role of viral load in the pathogenesis of chicken anemia virus. J Gen Virol. 2005;86:1327–33. 10.1099/vir.0.80599-0 15831943

[16] Imai K, Mase M, Tsukamoto K, Hihara H, Yuasa N. Persistent infection with chicken anaemia virus and some effects of highly virulent infectious bursal disease virus infection on its persistence. Res Vet Sci. 1999;67:233–8. 10.1053/rvsc.1999.0313 10607503

[17] Rijsewijk FAM, dos Santos HF, Teixeira TF, Cibulski SP, Varela APM, Dezen D, Discovery of a genome of a distant relative of chicken anemia virus reveals a new member of the genus *Gyrovirus.* Arch Virol. 2011;156:1097–100. 10.1007/s00705-011-0971-6 21442232

[18] dos Santos HF, Knak MB, de Castro FL, Slongo J, Ritterbusch GA, Klein TAP, Variants of the recently discovered avian gyrovirus 2 are detected in southern Brazil and the Netherlands. Vet Microbiol. 2012;155:230–6. 10.1016/j.vetmic.2011.09.021 22018524

